# A Single-Center Retrospective Review of Patients with Suspected Malignant Hyperthermia Susceptibility

**DOI:** 10.7759/cureus.44661

**Published:** 2023-09-04

**Authors:** Chris Edwards, Fred C Dooley, Sandra Gonzalez, Thomas M Austin, Nikolaus Gravenstein

**Affiliations:** 1 Anesthesiology, University of Florida College of Medicine, Gainesville, USA

**Keywords:** dantrolene, ryr1, genetic testing, caffeine halothane contracture test, malignant hyperthermia (mh)

## Abstract

Purpose

The diagnosis of malignant hyperthermia susceptibility (MHS) has significant implications for the perioperative period that may persist for generations. Anesthetic medication options are reduced, anesthetic workstations require preparation to reduce exposure to inhaled volatile anesthetics, and patients may be excluded from surgery at ambulatory centers. In this study, we sought to better characterize the etiology of MHS diagnoses in our health system and the downstream effects of this diagnosis on anesthetic care.

Methods

We retrospectively reviewed the electronic medical records of 55 patients with a documented concern for MHS who received care at University of Florida (UF) Health between 2014 and 2020. We characterized the etiology of the patient’s MHS diagnosis, whether this diagnosis was supported by formal genetic or muscle contracture testing, and the details of the recorded anesthetics that were delivered to these patients.

Results

The 55 patients with suspected MHS were evenly split between those with a family history of malignant hyperthermia (MH) (28/55) and those with a concern for MHS in their personal medical history (27/55). Of the 28 patients with a family history of MH, 16 reported that the affected family member was a first-degree relative, and two of these 16 reported that the affected family member had undergone confirmatory muscle contracture testing. Of the 27 patients with a personal history suspicious for MHS, two had undergone confirmatory genetic testing, and two patients had anesthetic records available for review where intraoperative MH was suspected and treated with dantrolene. An additional four patients were told of a concern about MHS due to another underlying diagnosis. No patients with a personal history suspicious of MHS had undergone confirmatory muscle contracture testing. These 55 patients underwent 87 anesthetics, and exclusively non-triggering anesthetic techniques were utilized in nearly all cases. In pediatric patients, some perioperative challenges were identified, related to the avoidance of mask inhalational induction. Only six of these 87 anesthetics occurred at our ambulatory surgery centers, a proportion (6.9%) lower than that of the general surgical population at UF Health (20.0%).

Conclusions

Among patients suspected to be MH susceptible in our health system over a six-year period, a minority (8/55) were supported by clear records of a prior MH event, confirmatory genetic or muscle contracture testing, or an underlying diagnosis closely linked to MH. The vast majority had limited documentation supporting their MH risk but continued to be treated with non-triggering anesthetics and were less likely to have surgery at an ambulatory surgery center than our overall surgical population. Among pediatric patients, some anesthetic challenges related to delivering non-triggering anesthetics were identified. Improving the documentation of index cases of MH and increasing referrals to clinical geneticists and genetic testing may be a viable route to decreasing the proportion of suspected MHS patients with a poorly characterized risk profile.

## Introduction

The diagnosis of malignant hyperthermia susceptibility (MHS) has significant implications for patients, their families, and the medical personnel who care for them. Some outpatient surgery centers exclude patients with a personal or family history of malignant hyperthermia (MH). Anesthetic medication options for MHS patients exclude inhaled volatile anesthetics and succinylcholine. The need to avoid inhaled volatile anesthetic inductions in MHS pediatric patients leads to alternative inductions that may cause added perioperative stress. Anesthesia personnel must also make special preparations for their anesthesia workstations, sometimes requiring machine flush times approaching two hours for institutions that do not have access to charcoal filters [1]. The anesthetic care of family members for subsequent generations may be similarly affected (with a baseline genetic prevalence of MH susceptibility of 1:2750 and predominately autosomal dominant inheritance [2], 11 generations from an MH susceptible proband are needed to reapproach the baseline population risk of MH!). Genetic and muscle contracture testing options exist to assess for MH susceptibility, but both have significant limitations [3], leading many individuals to be unsure of their MH risk.

We undertook a retrospective electronic medical record (EMR) review to better understand the complexities of diagnosing and caring for MHS patients at the University of Florida (UF) Health. We sought to delineate the most common ways that patients are acquiring a diagnosis of MHS in our health system, as well as the downstream effects of this diagnosis on their anesthetic care.

## Materials and methods

After approval from the University of Florida Institutional Review Board (IRB202202676), we requested an integrated data registry pull of any electronic patient records at the University of Florida (UF), Gainesville, USA, Health between April 2014 and June 2020 with either (1) the diagnosis of 'malignant hyperthermia’ or 'malignant hyperpyrexia’ listed in the problem list or (2) the specific term 'malignant hyperthermia’ found in any pre-anesthetic evaluation notes. This data query initially identified 112 patients. These records were manually reviewed by a study author (CE) with relevant experience as an MH Hotline consultant to determine whether there was objective evidence in the EMR of a true concern for MH susceptibility, or whether a statement such as ‘patient denies family history of malignant hyperthermia’ had inadvertently triggered inclusion in the initial list of patients. There were 57 such records where the words ‘malignant hyperthermia’ triggered inclusion in the data pull despite no evidence of a clinical concern for MHS. These records were excluded from further consideration. In the remaining 55 patient records, there was a clear concern for MH susceptibility.

**Figure 1:**
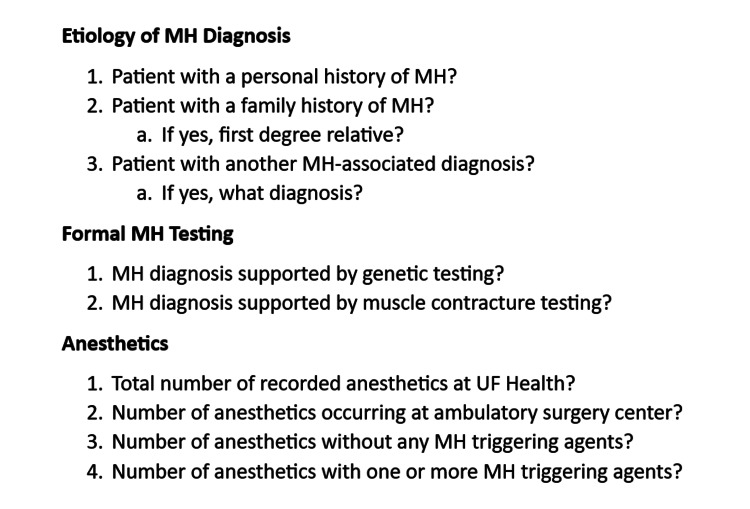
Malignant hyperthermia susceptibility chart review MH: malignant hyperthermia

Each of these 55 patients with concern for MHS had their perioperative records reviewed in detail, with particular attention to (1) why the patient was considered MHS, (2) whether this diagnosis was supported by formal genetic or muscle contracture testing, and (3) details of the anesthetics that were delivered to these patients. Specifically, the questions listed in Figure 1 were answered, as best as possible based on chart review, for all patients.

Additionally, any significant anesthetic challenges or complications deemed to be related to or possibly related to the patient's MH susceptibility were noted.

## Results

Part one: summary statistics

Of the 55 patients reviewed, 27 (49%) reported a personal clinical history suggestive of MHS. These histories ranged from strongly suggestive of MHS (prior anesthetic records available with dantrolene administered) to well-reasoned and possibly suggestive of MHS (deemed high risk by a neurologist in the setting of myalgias and baseline creatine kinase (CK) elevations) to the majority that were weakly suggestive of MH (told of a concern for MH during a prior anesthetic, limited clinical details offered, anesthetic records unavailable). Only two of these 27 patients had prior anesthetic records available for review that were strongly suggestive of a prior intraoperative MH event. One patient experienced severe hypercarbia following the administration of both sevoflurane and succinylcholine for retrograde urography and was treated with dantrolene. The second patient became hyperthermic and hemodynamically unstable during posterior spinal fusion following the administration of sevoflurane, was treated with dantrolene, and was subsequently found to have significant rhabdomyolysis with a CK value of 4,943 U/L.

Of the 55 patients reviewed, 28 (51%) reported a family history of MH. Of these, 16 reported that the affected family member was a first-degree relative, and two of these 16 reported that the affected family member had undergone muscle contracture testing that confirmed MHS.

Four patients reported MHS due to another diagnosis. These were congenital minicore myopathy, hyperkalemic periodic paralysis, Charcot-Marie-Tooth, and complex I mitochondrial disorder. It should be briefly noted here that only congenital minicore myopathy is actually strongly linked to MHS [4]. Hyperkalemic (and hypokalemic) period paralysis may also be associated with MHS [4,5]. However, there is no established elevated risk for MHS in Charcot-Marie-Tooth or most mitochondrial myopathies.

Confirmatory MHS testing was rarely found. Only two patients, both reporting a personal history of MH susceptibility, had undergone confirmatory genetic testing with diagnostic RYR1 abnormalities. None of our 55 patients reported having undergone a confirmatory muscle biopsy with contracture testing. However, as mentioned above, two affected family members (one patient’s son and another patient’s daughter) had undergone muscle contracture testing that confirmed MHS.

The above results are summarized in Table 1.

**Table 1:** Summary of the basis for malignant hyperthermia susceptibility MH: malignant hyperthermia; MHS: malignant hyperthermia susceptibility

Basis of patient’s malignant hyperthermia susceptibility	Number of patients (percentage)
Personal history of MHS	27/55 (49.1%)
Reports prior concern for MH event, records of event unavailable	17/55 (30.9%)
Reports MHS due to another diagnosis	4/55 (7.3%)
Prior recorded MH event with dantrolene administered	2/55 (3.6%)
Prior masseter muscle spasm/rigidity with succinylcholine	2/55 (3.6%)
MHS confirmed by genetic testing	2/55 (3.6%)
Family history of MHS	28/55 (50.9%)
First-degree relative without confirmatory testing	14/55 (25.5%)
Non first-degree relative without confirmatory testing	12/55 (21.8%)
First-degree relative confirmed by muscle contracture testing	2/55 (3.6%)

These 55 patients underwent 87 recorded anesthetics at UF Health. Malignant hyperthermia-triggering anesthetics were only utilized in four of these anesthetics. Two of these were index cases of intraoperative MH (the patient’s MHS was not known preoperatively), after which the patient was diagnosed with MH susceptibility, and future anesthetics utilized non-triggering agents. One patient who reported a possible history of masseter spasm with succinylcholine received an anesthetic utilizing maintenance volatile anesthesia, and one patient reporting a distant family history of MH (second or third cousin) received one anesthetic utilizing volatile anesthesia (but three other anesthetics utilizing exclusively non-triggering intravenous anesthetics). Only six of these 87 anesthetics (6.9%) took place at our ambulatory surgery centers, three of which were otolaryngology surgeries on the same pediatric patient. This compares to 63,378 of 317,558 anesthetics overall (20.0%) during the same six-year time period at UF Health that took place at ambulatory surgery centers.

Part two: individual adverse events

A small number of cases were identified where the patient’s reported MH susceptibility and the anesthesiology team’s desire to utilize a non-triggering anesthetic led to unique perioperative challenges. These are summarized as follows:

Case one: A two-year-old child weighing 13 kg with severe obstructive sleep apnea (OSA) presented for tonsillectomy and adenoidectomy. Due to a strong family history of MH, 6 mg oral Versed was administered preoperatively to facilitate intravenous (IV) access. After establishing access, propofol and remifentanil infusions were utilized for induction and maintenance of anesthesia. After a 77-minute anesthetic and in the setting of an apparent prolonged emergence, 0.1 mg of flumazenil was administered.

Case two: A three-year-old child weighing 17 kg presented for closed reduction and percutaneous pinning of an elbow fracture. Due to a reported family history of MH, 10 mg oral Versed and 70 mg oral ketamine were administered to facilitate IV access. When these oral medications proved inadequate for IV access, an additional 1 mg Versed and 50 mg ketamine were administered intramuscularly (IM).

Case three: An eight-year-old presented for three separate sclerotherapy procedures to treat a scapular vascular malformation. In the setting of second-degree family members with a reported history of MH, 67% nitrous oxide by mask was utilized to facilitate IV placement for the first of these anesthetics. During this nitrous oxide sedation for IV placement, the patient had an episode of emesis and gagging. Once the IV was placed, the team proceeded with rapid sequence induction using propofol and rocuronium.

Case four: A child underwent three anesthetics at our ambulatory surgery center: two sets of myringotomy tubes at one and two years of age, and an adenoidectomy at three years of age. Due to his mother’s reported history of MH, he received oral Versed, an IV catheter, and a propofol infusion for both sets of ear tubes rather than a more typical mask general anesthetic without an IV. During his third anesthetic for adenoidectomy at age three and weighing 19 kg, 70 mcg nasal dexmedetomidine and 50 mg IM ketamine were utilized to facilitate IV placement. Following an uneventful maintenance propofol infusion, the patient experienced laryngospasm at the time of extubation, which required rocuronium to resolve. This was subsequently reversed with sugammadex.

## Discussion

We present the above single-center retrospective review of patients with suspected MHS not because the results are particularly surprising, but rather because we think most anesthesiologists can relate and we suspect similar results could be found in most medical centers. In our study population, 55 patients were considered possibly MHS and treated as such during the vast majority of their anesthetics during a six-year period, yet the chart review found a strong case for MHS could only be made in eight of these 55 patients. Two patients had a prior suspected intraoperative MH event treated with dantrolene; two patients had prior genetic testing with a diagnostic ryanodine receptor (RyR1) abnormality; two patients had a first-degree relative with muscle contracture testing consistent with MHS; and two patients had underlying diagnoses either strongly linked (congenital minicore myopathy) or possibly associated (hyperkalemic periodic paralysis) with MHS. The remaining 47/55 patients treated as MHS had a variable and poorly characterized risk of a perioperative MH event that still carries an unacceptably high mortality rate approaching 10% [6].

In most other situations where a patient presents for non-urgent surgery with an elevated but not well-characterized risk of a major perioperative adverse event, we would consider further testing prior to proceeding with an anesthetic. Indeed, in a patient with elevated perioperative risk of a major adverse cardiac event that is not well characterized by functional status or prior cardiac studies, we are advised to consider further preoperative testing to better quantify risk and formulate our anesthetic plan [7]. But in the case of MHS, we are advised differently. The Malignant Hyperthermia Association of the United States (MHAUS) states that the care of MHS patients need not be restricted by the lack of formal MHS testing [8]. Similarly, the European Malignant Hyperthermia Group (EMHG) recommends that necessary surgery not be refused or postponed, regardless of the absence of definitive diagnostic testing [9]. The EMHG does, however, state that consultation with an MH expert may be valuable in possibly excluding MHS based on history. As individuals who frequently receive such inquiries through either the North American MH Hotline or the North American Malignant Hyperthermia Registry (NAMHR), such consultations are welcome but challenging. We frequently hear concerns about an elevated risk of MHS based on personal or family history, and similar to the results of our retrospective review, it is uncommon that the patient in question or their family members have undergone formal MHS testing, and even more infrequent that the patient has an underlying diagnosis clearly linked to MHS. While in some cases we can reassure a caller that a particular pathology has no established link to MHS and no special precautions need to be taken, often the situation is murky and the risk of MHS is possibly elevated based on history, and we recommend proceeding with a non-triggering anesthetic technique out of an abundance of caution.

We would suggest the difference between managing perioperative cardiac risk and MHS risk is primarily for two reasons. First, whenever we are presented with a patient with concern for MHS, we can simply opt for an anesthetic that avoids succinylcholine and volatile anesthetics, thereby eliminating the risk of pharmacologic triggering of MH. With charcoal breathing circuit filters commonly available at many medical centers, we can reduce exposure to volatile anesthetics to less than five parts per million in a matter of minutes, eliminating the need for prolonged machine flushes [10]. However, especially in the pediatric population, delivering non-triggering anesthetics is not without its own challenges. Not only were some of the anesthetics in our chart review more pharmacologically complicated than they would have been if there were no concern for MHS, but we would suggest that some of these children managed with intranasal and intramuscular medications likely experienced more perioperative stress than if they had been able to undergo a mask inhalational induction with sevoflurane.

Second, even if we were to seek further clarification of a patient’s MH susceptibility with formal testing, the current state of muscle contracture and genetic testing for MH has its own challenges. Only four centers in North America offer the caffeine halothane contracture test (CHCT) [11], a number that has decreased rather than increased in the past few years, and traveling to one of these centers may be time-consuming and prohibitively costly for patients. Additionally, CHCT requires an invasive surgical biopsy, often leading to multiple days of relative disability, and most centers have age and weight minimums due to the amount of quadriceps muscle that is harvested [12,13]. While genetic testing is more widely available, generally less costly, and certainly less invasive, this approach cannot definitively rule out a patient’s MH susceptibility. This is because the genetic basis of MH remains incompletely characterized, with up to 50% of confirmed MH-susceptible patients not carrying a known RYR1, calcium channel, voltage-dependent, L-type, alpha 1S subunit (CACNA1S), or SH3 and cysteine-rich domain-containing protein 3 (STAC3) variant [2]. Given this limited sensitivity of genetic testing, patients with personal or family history concerning MHS should generally continue to be treated with non-triggering anesthetics, even if genetic testing is performed and does not result in any known MHS variant.

With these limitations, what is the path forward in better characterizing MHS risk, and is there a way to more precisely identify the pool of patients that should be treated as MHS? We would offer a few suggestions:

First, when anesthesiologists identify a possible MH event, it is important that they not just inform the patient of this possibility but also document the particulars of the event. Including the intraoperative anesthetic record and/or pertinent laboratory values (blood gas, creatine kinase, urine myoglobin) in a letter to the patient (and copied in the EMR) will better inform future anesthesiologists of the patient’s possible MH event. A medical alert bracelet should also be considered, as this could prevent the administration of MH-triggering medications in an emergency, and MHAUS has partnered with the MedicAlert Foundation to offer these products [14]. Calculating the published MH clinical grading scale is another standardized method to characterize the likelihood of an MH event [15]. Some physicians might reasonably choose to proceed with a brief mask induction using sevoflurane to facilitate intravenous access in a pediatric patient with a relative who experienced a possible MH event if they were to find documentation that the event in question involved isolated intraoperative hyperthermia without evidence of rhabdomyolysis or hypercarbia and did not require dantrolene. When an intraoperative event suspicious for MH occurs, it is helpful to our understanding and management of MH to enroll the patient in the NAMHR. The patient can be enrolled anonymously by the provider or, once told of the event, self-enroll at anest.ufl.edu/namhr or call +1-352-2738911. Most patients enrolled in the NAMHR are self-enrolled. This helps build the database, which now exceeds 5,000 suspected MH cases in terms of their presentation and outcome.

Second, when a patient at elevated risk of MH is identified, we would recommend that the patient and family be offered a referral to a knowledgeable clinical geneticist. While anesthesiologists will often feel comfortable discussing clinical aspects of MH with patients, we suspect few are comfortable discussing the indications, risks, and benefits of genetic testing at the individual or family level. A geneticist can identify the proper tests, identify the family members who would most benefit from testing, discuss costs and possible implications for future insurance decisions or military enrollment, and ultimately interpret genetic variants. We have previously reported an infant who experienced a possible intraoperative MH event, one which resolved with prompt discontinuation of sevoflurane [16], and this infant and family benefitted from prompt inpatient consultation with clinical genetics. A genetic variant diagnostic for MH was quickly identified in the infant and his father; the family received proper counseling; and other siblings were set up for future genetic testing. The question of MHS was definitively resolved for this patient’s future anesthetics. Organizations like MHAUS and the NAMHR can assist with identifying such geneticists if one is not readily identified locally.

As discussed in a recent review article [17], ideally there will come a time when there is a highly sensitive physiologic test of MHS that is less invasive and more broadly available than current muscle contracture testing. As more patients with positive contracture testing continue to undergo comprehensive genetic testing, including whole exome sequencing, the genetic variants responsible for MHS will continue to be more completely characterized. Until then, recording and informing patients of the particulars of possible MH events and increasing the proportion of patients who are properly referred for genetic testing and consultation with clinical geneticists may help trim the proportion of patients who arrive for an anesthetic with a possible but poorly characterized MHS risk.

Our study has several limitations. First, as a single-center study with a limited number of patients, our results may not be widely applicable. There may be other medical centers where the risk profile of patients thought to be MHS is more fully interrogated, whether through more widespread referrals to confirmatory testing or simply through obtaining historical anesthetic records to evaluate purported index cases of MH. However, as several of our authors receive phone consultations regarding MHS risk from around the country, often with similarly limited histories and records, we believe it is unlikely that our institution is an outlier. Second, our patient population was limited to the results of a data registry request, and our analysis of the evidence supporting MH susceptibility was limited to an electronic chart review. Thus, it is likely that not all MHS patients at our institution were identified, and it is possible that patients and clinicians knew more information than was documented in the medical record. Finally, our analysis of the effect of MHS on access to ambulatory surgery is likely to be subject to confounding factors, as it is quite possible that some MHS patients were otherwise more medically complex and were scheduled at the main hospital for reasons unrelated to MH risk. We view our analysis as a starting point in the discussion of the need to better characterize MH risk and welcome larger and more methodologically rigorous studies.

## Conclusions

Among patients suspected to be MH susceptible in our health system over a six-year period, a minority (8/55) were supported by clear records of a prior MH event, relevant genetic or muscle contracture testing, or an underlying diagnosis closely linked to MH. The vast majority had limited documentation supporting their MH risk but continued to be treated with overwhelmingly non-triggering anesthetics and were approximately three times less likely to have surgery at an ambulatory surgery center than our general surgical population. Among pediatric patients, some anesthetic challenges related to delivering non-triggering anesthetics were identified. Improving the documentation of index cases of MH and increasing referrals to clinical geneticists and genetic testing may be a viable route to decreasing the proportion of suspected MHS patients with a poorly characterized risk profile.
